# Dr. Morton vs. Dr. Jackson—Sulphuric Ether

**Published:** 1853-01

**Authors:** 


*Dr.* MORTON *vs. Dr.* JACKSON.—Sulphuric Ether.

It is generally known by the profession that Dr. Jackson, a celebrated physician and chemist of Boston, set up a claim as the discoverer of "the anaesthetic or pain subduing properties of sulphuric ether, antagonistic to that of Dr. Morton. The profession are also aware that Dr. Morton took out a patent for the use of ether for such purposes, &c. &c. It appears that Dr. Jackson wished to get a portion of the profits resulting from this. But to make it look a *little* more *professional* he concluded to charge $500 for advice given to Dr. Morton on the use of the ether. This appearing too little, he afterwards claimed twenty-five per cent of the profits, and ultimately sold out for one dollar cash in hand paid. The profession are also aware, that Dr. Morton after having secured his patent, finally threw it up and gave free right of use to all, and since has made application to Congress for some remuneration. There has now been two reports to Congress on this subject. The last is now before us, and although very lengthy would well repay perusal. We might here say, however, that both reports are able; and fully sustain the claims of Dr. Morton. The last report, we presume, is drawn up by Dr. Bissell, of Illinois, chairman of the committee. We publish some eight pages of this; because it embodies;—first, Dr. Jackson's objections to the inquiry before Congress; and secondly, and mainly, because it gives us a brief and concise history of what is known on this subject since the days of Hippocrates to the present time. We give, also, the conclusion to which the committee have arrived; and we are free to confess, that the conclusion seems to us inevitable. We know that it bears very hard on Dr. Jackson; but the committee in their impartiality could not avoid this. The Physicians of the Massachusetts General Hospital have done the same.

The petition presented to Congress by Dr. Morton, the autograph copy of which we have, contains the names of many of the most eminent medical men of Boston.

We cannot but think that if Drs. Warren, Bigelow, Hayword, or any of such, had laid claim to the discovery on the same grounds which Dr. Morton has, Dr. Jackson would never have contested it as he has. The fact is, the credit of the discovery justly belongs to Dr. Morton as a dentist, and dentistry, as a science, must ever claim this as one of her trophies. There is much in Dr. Morton's memoir, presented to the Natural Academy of Science of France, which we should like to give; but the profession have had much of the same, as it regards general information, and our space will not permit.

We give below that portion of the report to which we have alluded.

The Select Committee to whom was referred the Memorial of Dr. William T. G. Morton, asking remuneration from Congress for the discovery of the anæsthetic or pain-subduing properties of sulphuric ether,

REPORT:

That upon the suggestion of the memorialist, that his claim to the discovery was contested by Dr. Charles T. Jackson, of Boston, the chairman addressed to him a letter, notifying him of the proceedings, and of the day when the committee would begin the investigation; advising him, that if he desired to do so, he was at liberty to contest Dr. Morton's application. The chairman received a statement from Dr. Jackson, in reply. Afterwards a memorial from Dr. Jackson was presented to the House, and referred to your committee. And on the 20th day of Dec. 1851, at a meeting held pursuant to notice, both parties appeared before your committee: Dr. Morton by his counsel, J. M. Carlisle, Esq., and Dr. Jackson by J. L. Hayes, Esq. In his paper Dr. Jackson presented objections to the inquiry, combining, in effect, a plea that the matter was *res judicata*, and a plea to the jurisdiction of Congress, which were discussed and considered, as preliminary to a general investigation. The objections are embodied in the following extracts.

After avering that the discovery was his, and his only, and that he alone gave it to the world, Dr. Jackson proceeded as follows:

"He, the undersigned, therefore, distinctly and unequivocally claims to be the sole and original discoverer of the anæsthetic and pain-subduing properties of sulphuric ether; and hereby communicates the fact to the Congress of the United States, and declares that his rights have been fully admitted by all the scientific societies that have examined into the claims of the numerous aspirants to the honor of the discovery, and that, in consequence of this result of investigations of all the claimant's pretensions, the National Academy of Sciences of France awarded to the undersigned "the Monthyon prize for the greatest medical discovery," and the Government of France awarded to him also the Cross of the Legion of Honor, and the King of Sweden the gold Medal of Merit. He, the undersigned, therefore, regards the question of discovery settled in the scientific world, and cannot but express his surprise," &c., &c.

And, again, he "begs that he may be put to no further trouble and expense in defending his scientific rights to the discovery."

And, again, he begs "that he may be allowed to pursue his studies and labors in peace, and not be compelled to spend his valuable time in waiting upon Congress, merely for the purpose of seeing that his rights suffer no detriment."

Your committee being unable to perceive the force of these objections, overruled them, and in the discharge of the duty imposed on them by the House, proceeded with the investigation. A mass of written and printed statements was offered by Dr. Jackson, tending to impeach the character of Dr. Morton, which the latter requested should be received, he being allowed time to produce rebutting evidence, and to adduce evidence on his part impeaching Dr. Jackson's character for veracity, and proving several cases in which he had claimed the inventions* of others as his own. This your committee rejected, deeming it wholly irrelevant to the subject committed to them by the resolution of the House, and leading to a long and laborious trial of many immaterial issues.

Their first enquiry was directed to the question whether a discovery had in fact been made, important to mankind, and in its importance and value to the American people worthy of national recognition and reward.

The alleged discovery consists in the total annihilation or prevention of pain in the most severe surgical operations, and obstetric cases, by the inhalation of the vapor of sulphuric ether. It is alleged also that the pain-destroying agent is innocent, producing no injurious consequences to the patient inhaling it. If this be true, and it be indeed a discovery, its national importance, its importance to the human race, is manifest.

Intense pain is regarded by mankind, generally, as so serious an evil that it would have been strange indeed if efforts had not been early made to diminish this species of suffering. The use of the juice of the poppy, henbane, mandragora, and other narcotic preparations, to effect this object by their deadening influence, may be traced back till it disappears in the darkness of a highly remote antiquity. Intoxicating vapors were also employed, by way of inhalation, to produce the same effects as drugs of this nature introduced into the stomach. This appears from the account given by Herodotus, of the practice of the Scythians, several centuries before Christ, of using the vapor of hemp seed as a means of drunkenness. The known means of stupefaction were very early resorted to, in order to counteract pain produced by artificial causes. In executions, under the horrible form of crucifixion, soporific mixtures were administered to alleviate the pangs of the victim. The draught of vinegar and gall, or myrrh, offered to the Savior in his agony, was the ordinary tribute of human sympathy extorted from the bystander by the spectacle of intolerable anguish.

That some lethean anodyne might be found to assuage the torment of surgical operations as they were anciently performed, cauterizing the cut surfaces, instead of tying the arteries, was not only a favorite notion, but it had been in

* Magnetic Telegraph, &c.

some degree, however imperfect, reduced to practice. Pliny, the naturalist, who perished in the eruption of Vesuvius, which entombed the city of Herculaneum, in the year 79, bears distinct and decided testimony to this fact.

"It has a soporific power," says he in his description of the plant known as the mandragora or circeius—"It has a soporific power on the faculties of those who drink it. The ordinary potion is half a cup. It is drunk against serpents, and *before cuttings and puncturings*, lest they should be felt." (*Bibitur et contra serpentes, et ante sectiones, punctionesque, ne sentiantur.*)

When he comes to speak of the plant *eruca*, called by us the rocket, he informs us that its seeds, when drank, infused in wine, by criminals about to undergo the lash, produce a certain callousness or induration of feeling, (*duritiam quandam contra sensum induere.*)

Pliny also asserts that the stone *Memphitis*, powdered and applied in a liniment with vinegar, will stupefy parts to be cut or cauterized, "for it so paralyzes the part that it feels no pain; *nec sentit cruciatum.*"

Dioscorides, a Greek physician of Cilicia, in Asia, who was born about the time of Pliny's death, and who wrote an extensive work on the materia medica, observes, in his chapter on mandragora—

1. "Some boil down the roots in wine to a third part, and preserve the juice thus procured, and give one cyathus of it in sleeplessness and severe pains, of whatever part; also, *to cause the insensibility*—to produce the anæsthesia (*poiein anaisthesian*) *of those who are to be cut or cauterized.*"

2. "There is prepared, also, besides the decoction, a wine from the bark of the root, three minæ being thrown into a cask of sweet wine, and of this three cyathi are given *to those who are to be cut or cauterized, as aforesaid;* for being thrown into a deep sleep, *they do not perceive pain.*"

3. Speaking of another variety of mandragora, called *morion*, he observes, "medical men use it also for those who are to be cut or cauterized."

Dioscorides also describes the stone Memphitis, mentioned by Pliny, and says that when it is powdered and applied to parts to be cut or cauterized, they are rendered, *without the slightest danger*, wholly insensible to pain. Matthiolus, the commentator on Dioscorides, confirms his statement of the virtues of mandragora, which is repeated by Dodoneus. "Wine in which the roots of mandragora has been steeped,"

says this latter writer, "brings on sleep, and appeases all pains so that it is given to those who are to be cut, sawed or burned, in any parts of their body, that they may not perceive pain."

The expressions used by Apuleius, of Madaura, who flourished about a century after Pliny, are still more remarkable than those already quoted from the older authors. He says, when treating of mandragora, "If any one is to have a member mutilated, burned or sawed, (*mutilandum, comburendum vel serrandum,*) let him drink half an ounce with wine, and *let him sleep till the member is cut away, without any pain or sensation,* (*et tantum dormiet, quousque abscindatur membrum aliquo sine dolore et sensu.*")

It was not in Europe and in Western Asia alone, that these early efforts to discover some letheon were made, and attended with partial success. On the opposite side of the continent, the Chinese, who have anticipated the Europeans in so many important inventions, as in gun powder, the mariner's compass, printing, lithography, paper money, and the use of coal, seem to have been quite as far in advance of the occidental world, in medical science. They understood, ages before they were introduced into christendom, the use of substances containing iodine for the cure of the goitre, and employed spurred rye, ergot, to shorten dangerously prolonged labor in difficult accouchments. Among the therapeutic methods confirmed by the experience of thousands of years, the records of which they have preserved with religious veneration, the employment of an anæsthetic agent to paralyze the nervous sensibility before performing surgical operations, is distinctly set forth. Among a considerable number of Chinese works on the pharmacopæia, medicine and surgery, in the National Library at Paris, is one entitled *Kou-kin-i-tong*, or general collection of ancient and modern medicine, in fifty volumes, quarto. Several hundred biographical notices of the most distinguished physicians in China are prefixed to this work. The following curious passages occur in the sketch of the biography of *Hoa-tho*, who flourished under the dynasty of *Wei*, between the years 229 and 230 of our era. "When he determined that it was necessary to employ acupuncture, he applied it in two or three places; and so with the *moxa*, if that was indicated by the nature of the affection to be treated. But if the disease resided in parts upon which the needle, the *moxa*, or liquid medicaments could not operate, for example, in the bones, or the marrow of the bones, in the stomach, or the intestines, *he gave* the patient a preparation of hemp, (in the Chinese language *mayo*,) and after a few moments he became as insensible as if he had been drunk or dead. Then, as the case required, he performed operations, incisions, or amputations, and removed the cause of the malady, then he brought together and secured the tissues, and applied liniments. After a certain number of days, the patient recovered, *without having experienced during the operation the slightest pain*. *Hoa-tho* has published, under the title of *Nei-tchao-thou*, anatomical plates, which exhibit the interior of the human body, which have come down to our times, and enjoy a great reputation."

It will be noticed that the agent employed by *Hoa-tho*, which he calls *ma-yo*, hemp medicine, and which is called in the annals of the latter *Hans*, *mafo-san*, or hemp essence powder, is the extract of the same plant mentioned by Herodotus, twenty-three centuries ago, the *canuabis Indica*, the *hashisch* of the Arabs, which is now extensively cultivated in Hindostan, for the purpose of manufacturing the substance called *Bhang*, to produce a peculiar species of intoxication, at first seductive and delicious, but followed in its habitual use by terrible effects upon the constitution.

Almost a thousand years after the date of the unmistakable phrases quoted from Apuleius, according to the testimony of William of Tyre; and other chroniclers of the wars for the rescue of the holy sepulchre, and the fascinating narrative of *Marco Polo*, a state of anæsthesia was induced for very different purposes. It became an instrument in the hands of bold and crafty impostors to perpetrate and extend the most terrible fanaticism that the world has ever seen.

The employment of anæsthetic agents in surgical operations, was not forgotten or abandoned during the period when they were pressed into the appalling service just described. In the thirteenth century, anæsthesia was produced by inhalation of an anodyne vapor, in a mode oddly forestalling the practices of the present day, which is thus described in the following passage of the surgical treatise of Theodoric, who died in 1298. It is the receipt for the "spongia somnifera," as it is called in the rubric:

"The preparation of a scent for performing surgical operations, according to Master Hugo. It is made thus: Take of opium and the juice of unripe mulberry, of hyoscyamus, of the juice of the hemlock, of the juice of the leaves of the mandragora, of the juice of the woody ivy, of the juice of the orest mulberry, of the seeds of lettuce, of the seed of the burdock, which has large and round apples, and of the water hemlock, each one ounce ; mix the whole of these together in a brazen vessel, and then in it place a new sponge, and let the whole boil, and as long as the sun on the dog days, till it (the sponge) consumes it all, and let it be boiled away in it. As often as there is need of it, place the same sponge into warm water for one hour, and let it be applied to the nostrils till he who is to be operated on, (*qui incidendus est*,) has fallen asleep ; and in this state let the operation be performed, (*et sic fiat chirurgia*.) When this is finished, in order to rouse him, place another dipped in vinegar, frequently to his nose, or let the juice of the roots of fenigreek be squirted into his nostrils. Presently he awakens."

A French physician, residing in the neighborhood of Toulouse, M. Dauriol, asserts that, in the year 1832, he employed a method analogous to that of Theodoric, and specifies five cases in which he succeeded in performing painless operations.

September 23, 1828, M. Girardin read a letter before the Academy of Medicine, addressed to his Majesty Charles X, by Mr. Hickman, a surgeon of London, in which this surgeon announces a means of performing the most delicate and most dangerous operations, without producing pain in the individuals submitted to them. This proceeding consists in suspending insensibility by the methodical introduction of certain gases into the lungs. Mr. Hickman had tested his proceedings by repeated experiments on animals.

Guy de Chauliac and Brunus, are the only authors on medicine and surgery, besides Theodoric, who during this period, allude to prophylactic agents to avert pain. It may be presumed, therefore, that their employment was not generally very successful. Probably bad effects, such as congestion and asphyxia, and sometimes ending in death, followed their unsuccessful empiricism. J. Cannappe, the physician of Francis I, in his work printed at Lyons in 1532, *Le Guidon pour les Barbiers et les Chirurgiens*, the Surgeon's and Barber's Guide, describes the method of Theodoric and his followers, as already given above, and adds: "*Les autres donnent opium a borie*, et fontmal, specialement s'il est jeune ; et le apercoivent, car ce est avec une grande bataille de vertu animale et naturelle. J'ai oui quilz encourent manie, et par consequent la mort."

Thus far had the superinduction of anæsthesia, as a preventive of pain, made its way into surgical practice in the middle ages; and even then it must have been most beneficial in its influence in diminishing the mass of human suffering. Down to the time when Ambrose Pare, in the sixteenth century, suggested the application of slender ligatures to bleeding arteries, to arrest the hemorrhage of surgical wounds, no other means were employed to stem the flow of blood after capital operations than by scorching over the raw surface with a red hot iron, or plunging it into boiling pitch, or applying other strong potential cauteries. "The horrors of the patient, and his ungovernable cries, the hurry of the operators and assistants, the sparkling of the (heated) irons, and the hissing of the blood against them, must have made terrible scenes," says Mr. John Bell;" and surgery must, in those days, have been a horrible trade."

Haller, Deneux, and Blandin, report cases of operations performed upon patients, under the influence of alcoholic intoxication, in obstetric and other cases without pain; and Richerard has suggested that this expedient should be employed in the management of dislocations difficult to be reduced. For obvious reasons it has not been adopted by the profession. Mesmerism, also, has been the subject of grave discussions, and of some extraordinary statements, in this connection; but, whatever may be thought of the individual cases certified by witnesses, it is not too much to say that it is not likely ever to become a remedy of general application.

Opium has in all ages been employed to assuage pain. Van Helmont calls it the specific gift of the Creator. Guy de Chauliac used it, and many surgeons have followed his example in their operations. Sassard, surgeon of the hospital *de la Charite*, strongly recommended this practice in the last century. But the irregular action of opium, the excitability which it sometimes occasions, its bad effects upon the digestive organs and the nervous system, and the length of time during which its influence remains, are decisive objections to this agent. Dr. Esdaille has recently experimented upon this subject at Calcutta, but the results are altogether unfavorable.

Van Frieten, Juvet, and Teden, have advised that mechanical compression should be employed to prevent pain in amputations, but this expedient proved but partially effectual, and has serious inconveniences which require it to be rejected without question.

The application of ice also will diminish pain under these circumstances. Baron Larry, after the battle of Eylau, found a remarkable insensibility in the wounded who suffered amputations, owing to the intense cold. The injury to the general health of the patient is not, however, compensated by the imperfect and uncertain success of this remedy.

After the great improvement brought about by the introduction of ligatures, the inducement to seek for a safe and effectual nepenthe, though still great, was vastly less than before. No practical advance deserving to be mentioned was made in this direction until the great discovery of the available effects of sulphuric ether.

This substance had been known since the thirteenth century. Its formation was accurately described by Valerius Cordus in the sixteenth century. Frobenius first designated it ether, and published an account of it in the philosophical transactions in 1730.

Its use as a medical agent, first alluded to by Valerius Cordus, and mentioned by Hoffman, Cullen, Alston, Lewis, and Monroe, and other writers of the last century, has long been familiarly known. The history of its use by inhalation commenced with the pamphlet published in 1795, by Richard Pearson; and several communications from the same Dr. Pearson are to be found in the work of Dr. Beddoes on Factitious Airs, published at Bristol, England, in 1796. The same work contains a letter from one of Dr. Thornton's patients, giving an account of his use of ether, by Dr. Thornson's advice, in a case of pectoral catarrh. He says, "it gave almost immediate relief both to the oppression *and pain* in the chest." On the second trial he inhaled two spoonsfull, with "immediate relief as before, and I very soon after *fell* asleep." In 1815, Nysten, in the Dictionary of Medical Sciences, speaks of the inhalation of ether as familiarly known for *mitigating pains* in cholic. For the last fifty years most therapeutic authors mention its use by inhalation in asthma, &c., as Duncan, Murray, Brande, Christison, Pereira, Thompson, Barbier, Wendt, Voght, Sundelin, &c. Effects analagous to intoxication, when ether is inhaled, are stated by American authors, as Godman, (1822,) Mitchell, (1832,) Professor Samuel Jackson, (1833,) Wood and Bache, (1834,) Miller, (1846, and early in that year.)

Dr. John C. Warren, in his work on Etherization, says: "The general properties of ether have been known for more than a century, and the effect of its inhalation, in producing exhiliration and insensibility, has been understood for many years, not only by the scientific, but by young men in colleges and schools, and in the shop of the apothecary, who have frequently employed it for these purposes."

About half a century since, Sir Humphrey Davy, who had acted as an assistant to Dr. Beddoes, in the commencement of his career, suggested the possibility that a pain-subduing gas might be inhaled, as follows: "As *nitrous oxide*, in its extensive operation, appears capable of destroying physical pain, it may probably be used with advantage during surgical operations in which no great effusion of blood takes place." *Researches on Nitrous Oxide*, p. 556. Upon this hint, Dr. Horace Wells, of Hartford, Connecticut, in the autumn of of the year 1844, experimented with *nitrous oxide gas*, in the extraction of teeth; but this gas being found on trial to be unavailable for the desired purposes, he abandoned his experiments in December, 1844, and tried none afterwards.

Late in the autumn of 1844, Dr. E. E. Marcy, of Hartford, Conn., as appears from his own affidavit and that of F. C. Goodrich, of Hartford, suggested to Dr. Wells to substitute sulphuric ether for nitrous oxide, and informed him of its known effects, and how to make it. Marcy "administered the vapor of rectified sulphuric ether in my [his] office to a young man; * * * and after he had been rendered insensible to pain, cut from his head an encysted tumor of about the size of an English walnut. The operation was entirely unattended with pain." Dr. Marcy concluded that nitrous oxide was more safe, equally efficacious, and more easily administered than ether, and therefore to be preferred, and retained that opinion to December, 1849.

Dr. E. R. Smilie, of Boston, in October, 1846, asserted that he had employed successfully an *etherial* tincture of opium to subdue pain under the knife. He states that he applied this tincture by inhalâtion in the spring of 1844; that he opened a serious abscess on the neck of the late Mr. John Johnson, while he was rendered unconscious of pain from the operation by this tincture.

The Paris Medical Gazette, of March, 1846, gives an account of remarkable experiments performed by M. Ducos, by ether, on animals, exhibiting most of the phenomena since witnessed in the human body. Sir Benjamin Brodie tried it on Guinea pigs, whom it put sleep and killed. He doubted its safety.

Notwithstanding this long series of efforts to procure a true nepenthe, the object still seems unattainable to the wisest and boldest members of the surgical profession. Velpeau, than whom no higher authority can be quoted, said, in 1839, "to avoid pain in surgical operations is a chimera which it is not allowable to pursue at the present day. The cutting instrument, and pain, in operative medicine, are two words which never present themselves singly to the mind of the patient, and of which we must necessarily admit the association." Orfila, in his Toxicology, declares absolute insensibility to pain under surgical operations by etherization, to be a discovery entirely new. Dr. J. C. Warren says, "The discovery of a mode preventing pain in surgical operations has been an object of strong desire among surgeons from an early period. In my surgical lectures I have almost annually alluded to it, and stated the means which I have usually adopted for the attainment of the object. I have also freely declared that, notwithstanding the use of very large doses of narcotic substances, this desideratum had never been satisfactorily obtained. The successful use of any article of the materia medica for this purpose, would therefore be hailed by me as an alleviation of human suffering." Finally, Sir Benjamin Brodie, in a discourse at St, George's Hospital, at so late a date as October 1, 1846, alluding to mesmerism, said, "There is no greater desideratum, either in medicine or surgery, than to have the means of allaying or preventing bodily pain, not only in surgical operations, but in other cases also, but there is good reason to apprehend that it has not been reserved for the revival of animal magnetism under a new name, to accomplish that *for which all physicians and surgeons have been looking in vain, from the days of Hippocrates down to the present time.*" Testimonials like these might be multiplied indefinitely, but the names already quoted are of those universally recognized on both continents as the most illustrious cultivators of medical science. The desideratum of which Brodie despaired on the 1st of October, 1846, had been found, and its efficacy demonstrated within twenty-four hours preceding the delivery of his lecture. And in a few days after, the tidings were borne with the full speed of steam across the Atlantic, and dispersed over Europe and Asia, which for two thousand years had been looking for it in vain.

This sketch of the progress of human knowledge as to the inhalation of sulphuric ether and its effects, and as to attempts to superinduce anæsthesia by various agents in ancient and modern times, though by no means scientifically complete, is sufficiently so for the purpose for which your committee have introduced it, to show what was and what was not known upon the subject previously to the investigations and experiments of this memorialist.

Your committee are satisfied, upon a full and careful examination of all the evidence before them, that until the 30th of September, 1846, it was not known that sulphuric ether might safely be inhaled in sufficient quantity to produce total insensibility to pain under the severest surgical operations. The safety of this agent, its certainty, its efficacy, are now established beyond question, and acknowledged by the whole scientific world. This great discovery, by far the noblest contribution which medical science has made to humanity within the present century, and with which, looking through all ages, no other except that of Jenner can take rank, sprung to light in the year 1846, in the State of Massachusetts; and the memorialist, Dr. Wm. T. G. Morton, claims it as his own.

Certain it is, he was the first who exhibited it to the world, and the only one who publicly used or claimed it, until after its reality and efficacy had been fully established. The honor of the discovery, therefore, must be awarded to him, unless some one show, by satisfactory evidence, an older and a bethe title. From the 30th of September, 1846, until the 2d day of January, 1847, during which time this discovery passed successfully, the *experimentum crucis*, Dr. Morton was in full, and sole, and undisputed possession. For a time, he held the operative agent as a secret, but at last disclosed it, by letter, to the faculty of the Medical Hospital at Boston, with a view to its trial, in what is called in surgery *a capital case.* It was not until some time after this trial had been made, and proved successful, that a claim was publicly set up by any one to the honor or a share in the honor of the discovery.

The account given by Dr. Morton of the circumstances which directed his mind to the investigation, is simple and natural, and in every step corroborated by some marked circumstance, proved by the testimony of one or more disinterested witnesses. A narrative such as his, so supported, goes far to sustain the title, which possession, undisputed for a time, would have given him. It was prepared by him, and presented to the Academy of Arts and Sciences at Paris, by M. Arago, in July 1847. Notwithstanding its length, we have thought proper to insert it entire.

Upon a full examination of the whole case so far as time and means were afforded to your committee, they have come to the conclusion—

1st. That Dr. Horace Wells did not make any discovery of the anæsthetic properties of the vapor of sulphuric ether, which he himself considered reliable, and which he thought proper to give to the world. That his experiments were confined to nitrous oxide, but did not show it to be an efficient and reliable anæsthetic agent, proper to be used in surgical operations and in obsterical cases.

For the rest your committee have come to the same conclusions that were arrived at by the Trustees of the Massachusetts General Hospital, at their meeting in January 1848, and reconsidered and confirmed in 1849, and adopted by the former committee of the House, viz:

2nd. That Dr. Jackson does not appear at any time to have made any discovery in regard to ether, which was not in print in Great Britain some years before.

3d. That Dr. Morton, in 1846, discovered the facts, before unknown, that ether would prevent the pain of surgical operations: and that it might be given in sufficient quantity to effect this purpose without danger to life. He first established these facts by numerous operations on teeth, and afterwards induced the surgeons of the hospital to demonstrate its general applicability and importance in capital operations.

4th. That Dr. Jackson appeared to have had the belief that a power in ether to prevent pain in dental operations would be discovered. He advised various persons to attempt the discovery. But neither they or he took any measures to that end; and the world remained in entire ignorance of both the power and safety of ether, until Dr. Morton made his experiments.

5th. That the whole agency of Dr. Jackson in the matter appears to consist only in his having made certain suggestions, which aided Dr. Morton to make the discovery—a discovery which had for some time been the object of his labors and researches.

